# Individual management of cervical cancer in pregnancy

**DOI:** 10.1007/s00404-015-3980-y

**Published:** 2016-01-04

**Authors:** Thomas Hecking, Alina Abramian, Christian Domröse, Tabea Engeln, Thore Thiesler, Claudia Leutner, Ulrich Gembruch, Mignon-Denise Keyver-Paik, Walther Kuhn, Kirsten Kübler

**Affiliations:** Department of Gynecology, Center for Integrated Oncology, University of Bonn, Sigmund-Freud-Strasse 25, 53127 Bonn, Germany; Department of Obstetrics and Prenatal Medicine, University of Bonn, Sigmund-Freud-Strasse 25, 53127 Bonn, Germany; Institute of Pathology, Center for Integrated Oncology, University of Bonn, Sigmund-Freud-Strasse 25, 53127 Bonn, Germany; Department of Radiology, Center for Integrated Oncology, University of Bonn, Sigmund-Freud-Strasse 25, 53127 Bonn, Germany

**Keywords:** Cervical cancer, Pregnancy, Treatment, Chemotherapy, Review, Case series

## Abstract

**Purpose:**

The management of cervical cancer in pregnancy persists to be challenging. Therefore, identification of factors that influence the choice of therapeutic management is pivotal for an adequate patient counseling.

**Methods:**

We present a literature review of 26 studies reporting 121 pregnancies affected by cervical cancer. Additionally, we add a retrospective case series of five patients with pregnancy-associated cervical cancer diagnosed and treated in our clinic between 2006 and 2013.

**Results:**

The literature review revealed that the therapeutic management during pregnancy varies according to the gestational age at diagnosis, while in the postpartum period no influence on the treatment choice could be detected. Also in our case series the choice of oncologic therapy was influenced by the gestational age, the wish to continue the pregnancy and the risks of delaying definitive treatment.

**Conclusions:**

There are no standardized procedures concerning the treatment of cervical cancer in pregnancy. Therefore, in consultation with the patient and a multidisciplinary team, an adequate individualized treatment plan should be determined.

**Electronic supplementary material:**

The online version of this article (doi:10.1007/s00404-015-3980-y) contains supplementary material, which is available to authorized users.

## Introduction

Cervical cancer (CC), comprising both squamous and glandular differentiation, is not only the fourth most frequent malignancy but also the fourth leading cause of cancer-related death in women worldwide. Among the malignant tumors of the cervix, squamous cell carcinoma (SCC) is the most common subtype and therefore characterizes the clinical and epidemiological picture of the disease. Although the tumor is virtually preventable by human papillomavirus (HPV) vaccination and effective screening strategies, its peak occurrence coincides with the prime reproductive years in non-compliant populations [1]. Accordingly, CC is one of the three most common pregnancy-associated cancer types with a crude incidence rate of 4 per 100,000 pregnancies [2]. Different data suggest that a diagnosis in pregnancy does not affect survival rates negatively [3]. However, these observations should be handled with care due to limited literature. The treatment of CC in pregnancy is complex. It has to take into consideration the optimal oncologic therapy as well as the preservation of the health of the fetus. Treatment options include conservative and surgical approaches based on tumor size, lymph node involvement, gestational age (GA) and the patient’s wish to continue the pregnancy [4]. To help provide a basis for treatment decisions, we here report our clinical management of five women diagnosed and treated with SCC during pregnancy. Additionally, we present a literature review focused on treatment options of gestational CC. We hypothesized that the GA at diagnosis might influence the choice of treatment and aimed to evaluate whether this might affect maternal outcome.

## Materials and methods

### Patients

We retrospectively reviewed the medical files of 84 pregnant women out of a total of approximately 2800 patients who presented at the outpatient department for genital dysplasia of Bonn University between 2006 and 2013. Patients were retrieved from the pathological database (PathoPro software, Institute for Medical Software, Saarbrücken, Germany) using the following search terms: ‘pregnancy’, ‘weeks of gestation’, ‘cervical dysplasia’ and ‘abnormal Pap smear’. Out of the cohort, five (5.95 %) patients with SCC were identified and included in this case series. Clinical information was gained from medical records; developmental charts were used to assess health outcome of newborns; follow-up data were updated until October 2015. In all women, colposcopy was performed using the photo and video colposcope 3MV (Leisegang, Berlin, Germany); image processing was carried out using the 3MV-Videology Viewer software 3.8.5.6 (Leisegang); application of 3 % acetic acid allowed the identification of cervical epithelial changes. Additionally, in all patients, colposcopy-directed biopsies of suspicious lesions were undertaken. Pap tests were evaluated according to the Munich nomenclature II; World Health Organization (WHO) criteria were used for histopathological diagnosis; tumor grade was determined based on the modified Broders’ classification [5]; tumors were staged clinically according to the International Federation of Gynecology and Obstetrics (FIGO) system [6]; the lymph node status was recorded separately.

### Literature search

A systematic computerized search was performed using PubMed and Web of Science (1995-2014) with the language being restricted to English. The PubMed query was conducted by combining the following MeSH (Medical Subject Headings) terms: ‘pregnancy complications/neoplastic/therapy’, ‘uterine cervical neoplasms’ and ‘carcinoma/squamous cell’; the MeSH keyword ‘trachelectomy’ was excluded. A similar strategy was applied to the Web of Science database using the following query: Title (TI) = (cervical cancer) AND TI = (pregnancy) AND Research Area (SU) = (Oncology) AND TS = (therapy) AND TS = (squamous) NOT Topic (TS) = (trachelectomy). Abstracts were explored for relevant information; full-text articles were used for further details. Authors were contacted if the complete manuscript could not be retrieved otherwise. Publications to be reviewed were selected by TH and KK. Case reports and retrospective trials were included; unpublished data were not accepted.

Thirty-seven studies met inclusion criteria. Three publications were excluded since they provided a review only [7–9]; two publications were excluded since they analyzed preneoplastic lesions [10, 11]; three publications were excluded since they did not provide original data to each patient [12–14]; two publications were excluded since we were unable to retrieve the whole publication [15, 16]; one publication was excluded since a successful pregnancy after the treatment of CC was reported [17]; 26 studies reporting 121 pregnancies [18–43] were used for the systematic review. The following data points were collected: baseline characteristics (age at diagnosis, GA at diagnosis), tumor characteristics (FIGO stage, histopathology), therapy (during pregnancy, in the postpartum period), obstetric characteristics (obstetric history, mode and GA of delivery, neonatal outcome) and maternal outcome. Our aim was to identify typical pattern and trends according to the GA at diagnosis. Therefore, patients were divided by their time point of tumor detection in early (<20 weeks (wks) GA) and late-diagnosed (≥20 wks GA) disease.

### Statistical analysis

The *F* test was used to analyze the assumption of equal variances; the unpaired two-tailed Student’s *t* test was used to compare differences in the following groups: age at diagnosis, GA at delivery and follow-up. For categorical variables the Chi-square test was used to investigate statistical significance of differences: FIGO stage, histopathology, therapy during pregnancy, therapy in the postpartum period, mode of delivery and status of maternal outcome. Additionally, Yates correction was performed. Results with a *p* value of <0.05 were considered to be significant.

## Results

### Case series

Five cases of SCC in pregnancy were identified during the study period. Median age at diagnosis was 32 years (range 30–37; Table 1). Risk factors included tobacco use and high-risk HPV positivity. Three patients did not participate in the screening program; two women had abnormal Pap smear results before getting pregnant. All patients were referred to a colposcopic examination during pregnancy. Diagnosis of SCC was made by biopsy in four pregnant women; in one patient, cervical intraepithelial neoplasia (CIN) III was detected in gravidity. However, in the postpartum period, the conization tissue showed malignant cells suggesting progression of the disease. Four biopsies were performed in the second and one in the third trimenon for the diagnosis of cancer. All Pap smears were without evidence of malignancy. All patients of our case series were diagnosed with early staged SCC (Table 2). Poor prognostic factors included the presence of lymph node metastasis in one patient, lymphovascular invasion (LVI) in two and low differentiation in three women.

**Table 1:** Baseline patient characteristics

Case	Age at diagnosis (yrs)	Risk factor		Preconception period		Gestation period			Maternal outcome	
		Tobacco use	HPV type	Regular screening	Pap smear before pregnancy	Pap smear in first/second/third trimenon	GA (wks) at colposcopy-directed biopsy	Histologic result of biopsy	Status	Follow-up (mths)
1	37	Yes	HR^a^	No	ND	IVb/ND/ND	18	SCC	NED	106.51
2	34	No	16	Yes	IIID	II/II/ND	32	SCC	NED	52.20
3	30	No	HR^a^	No	ND	IIID/IIID/IVa	26	CIN III^b^	NED	44.84
4	32	Yes	HR^a^	No	ND	IVa/ND/ND	16	SCC	NED	78.24
5	32	Yes	HR^a^	Yes	III	III/IVa/ND	20	SCC	NED	25.90

*GA* gestational age, *CIN* cervical intraepithelial neoplasia, *HPV* human papillomavirus, *HR* high risk, *mths* months, *NED* no evidence of disease, *ND* not determined, *SCC* squamous cell carcinoma, *wks* weeks, *yrs* years

^a^HPV types not specified

^b^Diagnosis of SCC was made by conization in the postpartum period

**Table 2:** Tumor characteristics

Case	FIGO Stage	Histopathology of squamous cell cancer					Therapy		
		Lymph node metastasis	LVI	BVI	Grade	Resection margins	Neoadjuvant chemotherapy during pregnancy (GA, wks)	Surgical treatment (GA, wks)	Adjuvant treatment in the postpartum period
1	IA1	ND	Absent	Absent	3	R0	Absent	Conization (21), CD and hysterectomy (35)	Absent
2	IA1	ND	Absent	Absent	2	R0	Absent	CD (36), conization in the postpartum period	Absent
3	IB1	Present	Present	Absent	2	R0	Absent	CD (40), conization followed by radical hysterectomy with pelvic lymphadenectomy in the postpartum period	Cisplatin-based radiochemotherapy and brachytherapy
4	IB1	Absent	Absent	Absent	3	R0	Absent	Radical hysterectomy with fetus in situ and pelvic lymphadenectomy (19)	Absent
5	IB1	Absent	Present	Absent	3	R0	4 cycles cisplatin, 20 mg/m^2^ KOF d1-3, q3w (23, 26, 29, 32)	CD and radical hysterectomy with pelvic lymphadenectomy (35)	Cisplatin-based radiochemotherapy

*CD* cesarean delivery, *BVI* blood vessel invasion, *GA* gestational age, *LVI* lymphovascular invasion, *ND* not determined, *R0* complete resection with microscopically negative margins, *wks* weeks

**Fig. 1:**
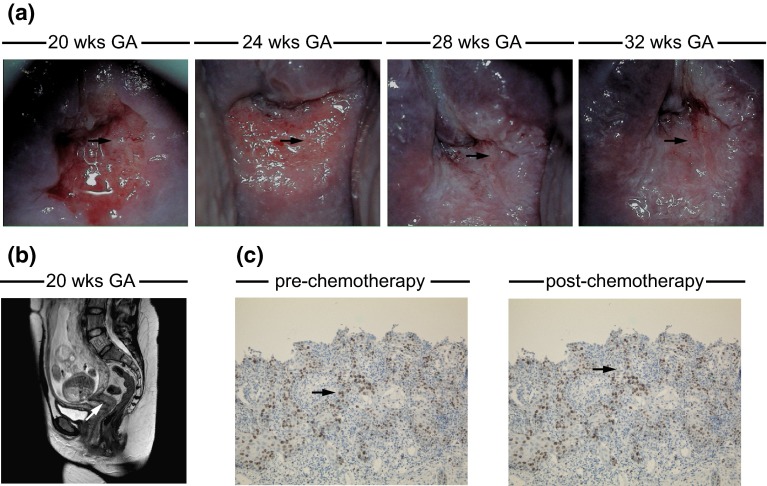
An example of the individual management of cervical cancer FIGO stage IB1 in pregnancy (case 5, after [44]). **a** Colposcopy in pregnancy was performed at initial diagnosis and subsequently during neoadjuvant chemotherapy to monitor the response to treatment; acetic acid was used for the visualization of cervical changes. Signs of invasive disease included atypical vessels and ulceration (*arrow*). **b** A pelvic MRI scan was performed at 20 wks GA and ruled out lymph node involvement; the sagittal T2-weighted image identifies the tumor by an increase in size and signal intensity (*arrow*). **c** Representative images of Ki-67 expression in non-treated and treated SCC visualized by immunohistochemistry (*brown*, *arrow*); hematoxylin (*blue*) was used for nuclear staining (bright field image, ×100 magnification)

Of all patients diagnosed with SCC in the second trimenon, one woman decided to terminate pregnancy (case 4). Radical hysterectomy with the fetus in situ and bilateral pelvic lymph node dissection was performed at the GA of 19 wks. Another woman was treated by conization at 21 wks of gestation (case 1). In this case, early staged SCC with negative margins for invasive disease was diagnosed. Pregnancy was prolonged, regular colposcopic controls were undertaken, and a cesarean delivery (CD) plus hysterectomy was performed at 35 wks of gestation. The final pathologic examination showed residual CIN but no invasive disease. The tumor of the third patient diagnosed in the second trimenon (20 wks GA) was 3 cm in diameter and exhibited LVI (case 5) [44]. Since this patient wished to continue her pregnancy, four cycles of neoadjuvant chemotherapy with cisplatin were administered. Clinical and colposcopic follow-ups were scheduled every 3 weeks confirming stable disease (Fig. 1a). An abdominal magnetic resonance imaging (MRI) scan at 20 wks of gestation was performed using a 1.5-T system (Intera, Philips Medical Systems, Best, Netherlands) to rule out lymph node involvement and advanced disease (Fig. 1b). Staging laparoscopy was refused by the patient. The fetal well-being was monitored regularly with ultrasonography and Doppler scan and showed no signs of intrauterine growth restriction. The patient tolerated chemotherapy well without any significant side effects. After reaching fetal pulmonary maturity, CD and radical hysterectomy with pelvic lymphadenectomy were performed at 35 wks of gestation. To assess the effect of chemotherapy on cell proliferation, 2–3 µm formalin-fixed paraffin-embedded tissue specimens were stained with the mouse antihuman Ki-67 IgG1 monoclonal antibody (clone MIB-1, dilution 1:500; Dako, Hamburg, Germany) using an automated staining system (Medac 480 S Autostainer; Medac, Wedel, Germany), HRP-conjugated goat anti-mouse IgG and the DAB system (Medac). The pathologic examination revealed a Ki-67 activity of 36.47 % after neoadjuvant chemotherapy compared to a proliferation index of 32.22 % at initial diagnosis suggesting stable disease (Fig. 1c). In the postpartum period, the patient received adjuvant treatment consisting of cisplatin-based radiochemotherapy.

In one patient, SCC was diagnosed in the early third trimenon (case 2). Due to a history of abnormal Pap smears over the past 5 years, she was referred to our colposcopic unit in early pregnancy. Cytologic results were normal, and accordingly, no colposcopy-directed biopsy was taken. However, the colposcopy in 32 wks of gestation showed a suspect area. Consequently, a biopsy was done revealing high-grade CIN with focal micro-invasive SCC. CD was performed at 36 wks of gestation; a conization was undertaken in the postpartum period.

In one case, SCC was diagnosed in the postpartum period (case 3). The patient was referred to our clinic 8 wks after delivery, and a conization was performed diagnosing invasive disease. Radical hysterectomy with pelvic lymph node dissection was undertaken. Additionally, the patient received adjuvant treatment consisting of cisplatin-based radiochemotherapy and brachytherapy.

With regard to the obstetrical history, the median gestational age at delivery was 35.5 wks (range 35–40; Table 3). All children were born with average birth weights for their GA. No significant complications at birth and neonatal course were noted. No long-term complications were detected. In particular, the child whose mother received platinum-based chemotherapy during pregnancy showed a normal development. As of today, all patients (Table 1) and their children (Table 3) undergo regular checkups and are healthy and alive.

**Table 3:** Obstetric characteristics

Case	Gravida/para/abortus	Delivery		Neonatal outcome			Long-term follow-up	
		GA (wks)	Mode	Weight (centile)	Apgar score	pH umbilical artery	Status	Age (yrs)
1	II/I/0	35	CD	2930 g (82)	7/8/9	7.36	Alive	8.43
2	I/0/0	36	CD	2660 g (61)	9/9/9	7.38	Alive	4.22
3	I/0/0	40	CD	3900 g (82)	5/9/10	7.23	Alive	4.07
4	I/0/0	19	Interruption	ND	ND	ND	ND	ND
5	II/I/0	35	CD	2525 g (56)	5/6/9	7.38	Alive	1.61

*CD* cesarean delivery, *GA* gestational age, *wks* weeks, *yrs* years

### Review of the literature

Details for each case of the literature review are given in Supplementary Table S1. Tumors were classified according to their GA at detection in early and late-diagnosed disease, and variables were compared (Table 4). No difference was found between groups regarding patient and tumor characteristics as well as maternal outcome. We identified a significant discrepancy in the oncological management during pregnancy but not after delivery between early- and late-diagnosed tumors. Accordingly, the GA and the mode of delivery differed in both groups.

**Table 4:** Variables extracted from the literature review grouped according to the gestational age

GA at diagnosis (wks)	Patient characteristics	Tumor characteristics		Therapy		Obstetric characteristics		Maternal outcome	
	Age at diagnosis (yrs)	FIGO stage	Histopathology	During pregnancy	Postpartum period	GA (wks) of delivery	Mode of delivery	Follow-up (mths)	Status
(*n*)*	Median (range)	(*n*)	(*n*)	(*n*)**	(*n*)**	Median (range)	(*n*)	Median (range)	(*n*)
<20 (60)	32 (23–47)	IA (16), IB (34), IIA (4), IIB (6), IIIB (0), IVB (0)	SCC (42), Non-SCC (13), ND (5)	None (12), IR (20), ChT (14), C (20), RT (2), pL (18)	None (5), CD/RH or TH ± RT/ChT (23)****, CD/TR (4), RH or TH ± RT/ChT (23), RT ± ChT (4), C (1)	33 (7–41)	VD (6), CD (29), IR (22), ND (3)	56.5 (1–248)	NED (50), DOD (3), RD (2), ND (5)
≥20 (44)	34 (24–41)	IA (9), IB (28), IIA (3), IIB (1), IIIB (2), IVB (1)	SCC (33), Non-SCC (4), ND (7)	None (21), IR (3), ChT (13), C (10), RT (0), pL (9)	None (3), CD/RH or TH ± RT/ChT (10)****, CD/TR (1), RH or TH ± RT/ChT (28), RT ± ChT (1), C (1)	35 (21–41)	VD (3), CD (34), IR (3), ND (4)	32 (3–147)	NED (39), DOD (4), RD (1), ND (0)
*p* value***	n.s.	n.s.	n.s.	<0.02	n.s.	<0.0001	<0.01	n.s.	n.s.

*C* conization, *CD* cesarean delivery, *ChT* chemotherapy, *DOD* dead of disease, *GA* gestational age, *IR* interruption, *mths* months, *NED* no evidence of disease, *ND* not determined, *n.s.* not significant, *pL* pelvic lymphadenectomy, *RH* radical hysterectomy, *RD* recurrent disease, *RT* radiotherapy, *SCC* squamous cell carcinoma, *TH* total hysterectomy, *TR* trachelectomy, *wks* weeks, *yrs* years, *VD* vaginal delivery

* Cases diagnosed in the preconception and the postpartum period are excluded

** Includes multiple listings of cases

*** After Yates correction

**** Includes cases in which ChT or RT were performed alone

## Discussion

Although its incidence rate has been declining in the last years, CC is one of the most frequently diagnosed malignancies in pregnancy. Due to an increase in women choosing to become pregnant later in their lives, even a further rise of gestational CC is plausible [2]. Literature on the clinical management of CC in pregnancy is scarce. Therefore, we retrospectively analyzed pregnancy-associated CC cases in our clinic and performed a literature review that focused on treatment approaches.

We found therapeutic modalities in pregnancy still to be limited consisting mainly of conization and chemotherapy. Alternatively, tumors can either be followed up or pregnancy can be interrupted for definitive therapy according to guidelines [45]. Treatment decisions in pregnancy depended on the GA at first diagnosis. Accordingly, women obtained interruptions until 20 wks after conception more often as women who are more than 20-wk pregnant. In the latter case, the concept of ‘watchful waiting’ appeared to be typical. According to these results, also the mode and the GA at delivery correlated with the time point of first diagnosis in pregnancy. However, different treatment approaches across all gestational ages appeared not to affect negatively the mother’s survival. Taken together, our literature review underlines the therapeutic complexity of CC in pregnant women since decisions have to take into account the impact upon mother and fetus. Consistent with this observation, different treatment approaches were also seen in our case series of pregnancy-associated SCC.

**Fig. 2:**
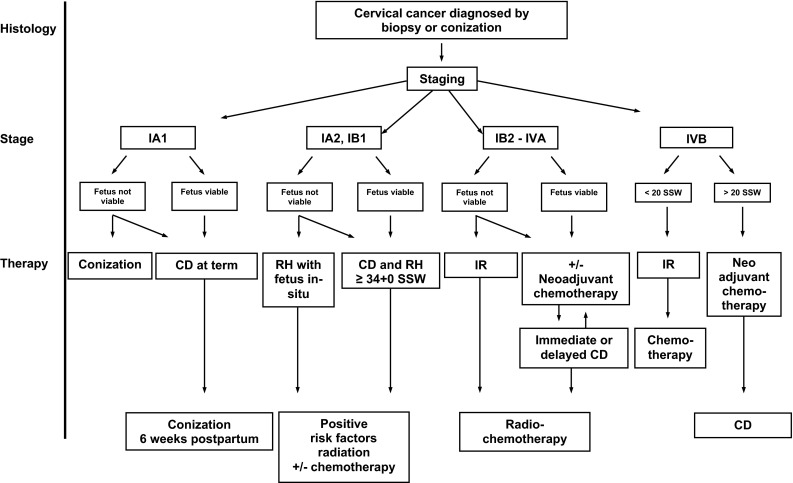
Therapeutic algorithm for cervical carcinoma in pregnancy (after [46, 47]). Scheme for the treatment options in women with gestational cervical cancer according to the FIGO stage (*IR* interruption, *CD* cesarean delivery, *RH* radical hysterectomy)

An overview of treatment options according to the clinical stage is given in Fig. 2 modified after Hunter et al. [46, 47]. Prolongation of the pregnancy at an early stage and thus delaying definite treatment were reported to be safe [3]. Diagnostic conization, though associated with a significant risk of hemorrhage, might help to assess the actual invasion depth after biopsy showing micro-invasive disease [4]. Lymph node involvement can be assessed by MRI scans, thereby providing the basis for prolonging pregnancy [22]. Alternatively, laparoscopic lymphadenectomy has emerged as an effective and more precise procedure during pregnancy [48]. Neoadjuvant chemotherapy during gestation might be possible in selected patient groups, defined by advanced disease or high-risk carcinomas, allowing for fetal maturation. However, the choice has to be individually made, weighing the risk of antenatal toxicity against the delay of curative treatment. Platinum was shown to be a safe option during pregnancy [49], especially since platinum concentrations are extremely low in the fetal unit suggesting placental filtration [50]. The timing of delivery is a critical point in the management of gestation-associated CC. In accordance with current guidelines, preterm labor was tolerated in our cohort to compromise between fetal maturity and completion of the mothers’ oncological treatment [51].

Undoubtedly, the most successful strategy against CC in pregnancy is the participation in preconceptive cancer screening programs. Most of the patients in our cohort did not undergo regular gynecological examinations before getting pregnant. Since the Pap smear is an essential part of early antenatal care, it allows detecting cervical changes also in the under-screened population and has the advantage to detect a tumor at an early stage. Accordingly, all CCs in the non-screened patients of our case series were diagnosed at FIGO stage I, which is in accordance with published data [52]. Only two patients of our cohort participated regularly in the screening program. They were diagnosed with suspect Pap smears before getting pregnant and referred for colposcopy only after the onset of pregnancy. Their subsequent diagnosis of malignancy underlines the importance of the histopathological evaluation in the case of repeated abnormal cytological results. Especially during pregnancy, colposcopy-directed biopsies should be preferred since hormone-related cellular changes may be misidentified in Pap smears [53]. Colposcopy-directed biopsy is a safe and reliable procedure during pregnancy. Delayed bleeding can occur but is often successfully resolved with the application of pressure [54].

Taken together, women with suspect Pap test desiring to bear a child should postpone their pregnancy until definite treatment of dysplasia took place. Pregnant women with suspect Pap smears should be referred to a colposcopic unit where experienced colposcopists are familiar with the physiological changes of the uterine cervix during pregnancy. Being diagnosed with CC during pregnancy, management depends on different factors including the stage of disease, the week of gestation and the woman’s desire to bear a child. Thus, an individualized treatment plan for each patient is required.

## Electronic supplementary material

Below is the link to the electronic supplementary material.

Supplementary material 1 (DOCX 124 kb)
