# Supplementary material for: Individual management of cervical cancer in pregnancy
Source: Arch Gynecol Obstet. 2016 Jan 4;293:931–9. doi: 10.1007/s00404-015-3980-y (PMC4829625; doi:10.1007/s00404-015-3980-y)
Supplement: Supplementary file 1 — Supplementary material 1 (DOCX 124 kb) [file 404_2015_3980_MOESM1_ESM.docx]

**Supplementary Table S1. Review of the literature**

|  |  |  |  |  |  |  |  |  |  |  |  |  |  |
| --- | --- | --- | --- | --- | --- | --- | --- | --- | --- | --- | --- | --- | --- |
| **Reference** | **Case** | **Patient characteristics** | | **Tumor characteristics** | | **Therapy** | | **Obstetric characteristics** | | | | **Maternal outcome** | |
|  |  | **Age at diagnosis (yrs)** | **GA at diagnosis (wks)** | **FIGO stage** | **Histo-pathology** | **During pregnancy** | **Postpartum period** | **Gravida / para** | **GA (wks) of delivery** | **Mode of delivery** | **Neonatal outcome** | **Status** | **Follow-up (mths)** |
| Allen DG et al., 1995 | 1 | 34 | 9 | IA1 | A | IR | RH | ND | 10 | IR | Abort | NED | 89 |
|  | 2 | 27 | 25 | IA1 | A/SCC | C | None | ND | 35 | ND | Alive | NED | 72 |
|  | 3 | 34 | 32 | IA1 | SCC | C | None | ND | 39 | ND | Alive | NED | 24 |
|  | 4 | 31 | 21 | IA1 | SCC | C | None | ND | 36 | ND | Alive | NED | 63 |
|  | 5 | 31 | 9 | IA1 | SCC | C | None | ND | 40 | ND | Alive | NED | 90 |
|  | 6 | 36 | 16 | IA1 | SCC | C | None | ND | 39 | ND | Alive | NED | 1 |
|  | 7 | 35 | 30 | IA1 | SCC | None | C | ND | 40 | ND | Alive | NED | 44 |
|  | 8 | 34 | PP | IA1 | SCC | None | C | ND | 39 | ND | Alive | NED | 2 |
|  | 9 | 24 | PP | IA1 | SCC | None | C | ND | 41 | ND | Alive | NED | 113 |
|  | 10 | 37 | PP | IA1 | A | None | C, RH | ND | 8 | ND | Abort | NED | 46 |
|  | 11 | 36 | PP | IA1 | SCC | None | C, RH | ND | 39 | ND | Alive | NED | 47 |
|  | 12 | 38 | PP | IB | SCC | C | CD/RH | ND | 34 | CD | Alive | NED | 69 |
|  | 13 | 28 | 16 | IB | SCC | C | CD/RH | ND | 35 | CD | Alive | NED | 62 |
|  | 14 | 41 | 38 | IB | SCC | None | RH | ND | 38 | CD | Alive | RD | 20 |
|  | 15 | 31 | 21 | IIA | SCC | None | RH | ND | 40 | VD | Alive | NED | 88 |
|  | 16 | 36 | PP | IB | A/SCC | None | RH | ND | 40 | ND | Alive | NED | 115 |
|  | 17 | 27 | PP | IB | SCC | None | RH | ND | 40 | ND | Alive | NED | 112 |
|  | 18 | 33 | PP | IB | SCC | None | RH | ND | 40 | ND | Alive | NED | 48 |
|  | 19 | 35 | PP | IB | SCC | None | RH | ND | 40 | ND | Alive | NED | 96 |
|  | 20 | 34 | PP | IB | A | None | RH, RT | ND | 40 | ND | Alive | NED | 48 |
|  | 21 | 38 | PP | IB | A | None | RH | ND | 40 | ND | Alive | NED | 7 |
|  | 22 | 27 | PP | IIIB | SCC | None | TH, RT/ChT | ND | 40 | ND | Alive | NED | 153 |
| Sorosky JI et al., 1995 | 23 | 29 | 18 | IB1 | SCC | None | CD/RH | 4 / 3 | 33 | CD | Alive | NED | 68 |
|  | 24 | 42 | 8 | IB1 | SCC | None | CD/RH | 4 / 3 | 38 | CD | Alive | NED | 60 |
|  | 25 | 28 | 16 | IB1 | SCC | None | CD/RH, RT | 1 / 0 | 37 | CD | Alive | NED | 43 |
|  | 26 | 36 | 21 | IB1 | SCC | None | CD/RH | 2 / 1 | 37 | CD | Alive | NED | 42 |
|  | 27 | 36 | 28 | IB1 | SCC | None | CD/RH | 4 / 3 | 35 | CD | Alive | NED | 23 |
|  | 28 | 26 | 34 | IB1 | SCC | None | CD/RH | 1 / 0 | 37 | CD | Alive | NED | 21 |
|  | 29 | 27 | 18 | IB1 | SCC | None | CD/RH | 3 / 2 | 36 | CD | Alive | NED | 19 |
|  | 30 | 31 | PC | IB1 | SCC | None | CD/RH | 7 / 2 | 37 | CD | Alive | NED | 13 |
| Tewari K et al., 1998 | 31 | 34 | 23 | IB2 | SCC | None | CD/RH | 6 / 5 | 33 | CD | Alive | NED | ND |
| Tewari K et al., 1998 | 32 | 34 | 16 | IIA | SCC | 3 cycl Vin/Cis, 3 cycl Cis | CD/RH, RT/ChT | 5 / 1 | 34 | CD | Alive/NICU | RD | 5 |
|  | 33 | 36 | 21 | IB2 | SCC | 4 cycl Vin/Cis | CD/RH | 1 / 0 | 32 | CD | Alive | NED | 24 |
| van Vliet W et al., 1998 | 34 | ND | 34 | IIA | ND | None | RT | ND | 36 | CD | Alive/NICU | NED | 142 |
|  | 35 | ND | 26 | IB | ND | None | CD/RH | ND | 32 | CD | Alive/NICU | NED | 106 |
|  | 36 | ND | 28 | IB | ND | None | CD/RH, RT | ND | 32 | CD | Alive/NICU | NED | 92 |
|  | 37 | ND | 40 | IB | A | None | RH, RT | ND | 40 | VD | Alive | DOD | 14 |
|  | 38 | ND | 32 | IB | ND | None | CD/RH | ND | 35 | CD | Alive/NICU | NED | 54 |
|  | 39 | ND | 23 | IB | ND | None | CD/RH | ND | 33 | CD | Alive/NICU | NED | 16 |
|  | 40 | ND | 16 | IB | ND | IR | RH | ND | 17 | IR | Abort | NED | 248 |
|  | 41 | ND | 12 | IB | ND | IR | RH, RT/ChT | ND | 13 | IR | Abort | NED | 192 |
|  | 42 | ND | 15 | IB | ND | IR | RH, RT | ND | 16 | IR | Abort | DOD | 68 |
|  | 43 | ND | 6 | IIB | ND | IR | RH, RT/ChT | ND | 7 | IR | Abort | NED | 130 |
|  | 44 | ND | 20 | IB | ND | IR | RH | ND | 21 | IR | Abort | NED | 102 |
|  | 45 | ND | 11 | IB | ND | IR | RH | ND | 12 | IR | Abort | NED | 48 |
| Marana HR et al., 2001 | 46 | 26 | 15 | IIB | SCC | 2 cycl Bleo/Cis | None | 4 / 3 | 38 | CD | Alive | DOD | 19 |
| Takushi M et al., 2002 | 47 | 33 | 14 | IA1 | SCC | IR | TH | 4 / 2 | 17 | IR | Abort | NED | 69 |
|  | 48 | 32 | 37 | IA2 | SCC | None | CD/RH | 3 / 2 | 39 | CD | Alive | NED | 114 |
|  | 49 | 34 | 6 | IA2 | SCC | IR | RH | 5 / 2 | 9 | IR | Abort | NED | 87 |
|  | 50 | 25 | PP | IB1 | A | None | RH | 3 / 1 | ND | ND | Alive | NED | 88 |
|  | 51 | 34 | 20 | IB1 | SCC | IR | RH | 4 / 2 | 21 | IR | Abort | NED | 70 |
|  | 52 | 39 | 13 | IB1 | SCC | IR | RH | 5 / 3 | 15 | IR | Abort | NED | 53 |
|  | 53 | 38 | 7 | IB1 | SCC | IR | RH | 7 / 4 | 9 | IR | Abort | NED | 27 |
|  | 54 | 32 | 9 | IB2 | SCC | IR | RH, RT | 4 / 2 | 9 | IR | Abort | NED | 93 |
|  | 55 | 34 | PP | IB2 | SCC | None | RH | 3 / 1 | ND | ND | Alive | NED | 85 |
|  | 56 | 38 | 13 | IB2 | SCC | IR | RH, RT | 5 / 1 | 17 | IR | Abort | NED | 43 |
|  | 57 | 42 | 6 | IIA | SCC | IR | RH, RT | 9 / 5 | 9 | IR | Abort | NED | 120 |
|  | 58 | 31 | 13 | IIB | SCC | IR | RH, RT | 5 / 2 | 15 | IR | Abort | NED | 103 |
|  | 59 | 39 | 9 | IIB | SCC | IR | RH, RT | 4 / 3 | 11 | IR | Abort | NED | 97 |
|  | 60 | 40 | 11 | IIB | SCC | IR | RH, RT | 5 / 2 | 12 | IR | Abort | NED | 82 |
|  | 61 | 34 | PP | IIIB | SCC | None | RT | 2 / 1 | ND | ND | Alive | NED | 101 |
|  | 62 | 30 | 29 | IIIB | SCC | None | RT | 3 / 2 | 30 | CD | Alive | DOD | 4 |
|  | 63 | 32 | 16 | IA1 | SCC | None | RH | 5 / 3 | 37 | VD | Alive | NED | 156 |
|  | 64 | 30 | 27 | IA1 | SCC | None | CD/RH | 4 / 3 | 36 | CD | Alive | NED | 147 |
|  | 65 | 32 | 19 | IA1 | SCC | None | TH | 3 / 2 | 38 | VD | Alive | NED | 142 |
|  | 66 | 38 | 22 | IA1 | SCC | None | TH | 3 / 2 | 41 | VD | Alive | NED | 140 |
|  | 67 | 28 | 12 | IA1 | SCC | None | C | 5 / 1 | 37 | VD | Alive | NED | 101 |
|  | 68 | 37 | 10 | IA1 | SCC | None | TH | 6 / 3 | 39 | VD | Alive | NED | 82 |
|  | 69 | 27 | 18 | IA1 | SCC | None | TH | 2 / 1 | 34 | VD | Alive | NED | 70 |
|  | 70 | 33 | 16 | IA1 | SCC | None | TH | 5 / 3 | 40 | VD | Alive | NED | 52 |
|  | 71 | 35 | 20 | IA2 | SCC | None | CD/RH | 2 / 1 | 33 | CD | Alive | NED | 110 |
|  | 72 | 31 | 18 | IB1 | SCC | None | CD/RH | 3 / 2 | 31 | CD | Alive | NED | 97 |
|  | 73 | 32 | 17 | IB1 | SCC | None | CD/RH | 2 / 1 | 32 | CD | Alive | NED | 108 |
|  | 74 | 24 | 26 | IB2 | SCC | None | CD/RH, RT | 2 / 1 | 32 | CD | Alive | NED | 102 |
| Ostrom K et al., 2003 | 75 | 28 | 15 | IB2 | SCC | RT | RT | 4 / 3 | 20 | IR | Abort | NED | 12 |
|  | 76 | 35 | 15 | IB2 | SCC | RT | RT | 5 / 4 | 21 | IR | Abort | NED | 12 |
| Hogg R et al., 2005 | 77 | 40 | 27 | IB2 | SCC | None | CD/RH | ND | 32 | CD | Alive | NED | ND |
| Caluwaerts et al., 2006 | 78 | 28 | 15 | IB1 | SCC | 6 cycl Cis | CD/RH | 2/1 | 32 | CD | Alive | NED | 10 |
| Traen K et al., 2006 | 79 | 38 | 19 | IIA | SCC | 4 cycl Vin/Cis | CD/RH, ChT** | 1 / 0 | 33 | CD | Alive/NICU | NED | 80 |
| Bader AA et al. 2007 | 80 | 37 | 14 | IB1 | SCC | C | CD/RH | 1 / 0 | 32 | CD | Alive | NED | 17 |
| Karam A et al, 2007 | 81 | 28 | 23 | IB2 | SCC | Cis weekly (7 wk) | CD/RH, RT/ChT | 1 / 0 | 33 | CD | Alive | NED | 14 |
| Palaia I et al, 2007 | 82 | 30 | 19 | IIB | SCC | 3 cycl Cis | CD/RH | ND | 35 | CD | Alive | NED | 10 |
| Benhaim Y et al., 2008 | 83 | 31 | 22 | IIIB | SCC | 2 cycl Cis | RT/ChT | 3 / 1 | 28 | CD | Alive | DOD | 10 |
| González Bosquet E et al., 2008 | 84 | 28 | 25 | IB | SCC | None | CD/RH | 4 / 3 | 33 | CD | Alive | NED | 12 |
| Chun KC et al., 2010 | 85 | 27 | 26 | IB1 | ND | 3 cycl Cis/Pac | CD/RH | ND | 36 | CD | Alive | DOD | 49 |
|  | 86 | 32 | 29 | IIA | SCC | 1 cycl Cis/Pac | CD/RH | ND | 34 | CD | Alive | NED | 32 |
|  | 87 | 27 | 29 | IB2 | SCC | 1 cycl Cis/Pac | CD/RH, ChT** | ND | 37 | CD | Alive | NED | 60 |
| Favero et al., 2010 | 88 | 29 | 23 | IB2 | A | C/pL | CD/RH | ND / 1 | 35 | CD | Alive | NED | 68 |
|  | 89 | 40 | 15 | IB1 | A | IR/pL | RH, RT/ChT | ND / 2 | ND | IR | Abort | NED | 128 |
|  | 90 | 32 | 14 | IB1 | SCC | C/pL | CD/RH | ND / 0 | 32 | CD | Alive | NED | 102 |
|  | 91 | 36 | 21 | IB1 | A | IR/pL | RH | ND / 0 | ND | IR | Abort | NED | 68 |
|  | 92 | 34 | 22 | IA1 | SCC | C/pL | CD/RH | ND / 2 | 33 | CD | Alive | NED | 32 |
|  | 93 | 29 | 23 | IB1 | SCC | C/pL | CD/TR | ND / 0 | 33 | CD | Alive | NED | 18 |
|  | 94 | 29 | 12 | IB1 | A | C/pL | CD/TR | ND / 1 | 34 | CD | Alive | NED | 30 |
|  | 95 | 29 | 14 | IB1 | SCC | C/pL | CD/TR | ND / 0 | 34 | CD | Alive | NED | 10 |
|  | 96 | 35 | 14 | IB1 | A | C/pL/2-3 cycl Cis | CD/RH | ND / 1 | 33 | CD | Alive | NED | 12 |
|  | 97 | 26 | 6 | IA2 | A | C/pL | CD/TR | ND / 0 | 34 | CD | Alive | NED | 8 |
|  | 98 | 31 | 18 | IB1 | SCC | C/pL/2-3 cycl Cis | CD/RH | ND / 1 | 34 | CD | Alive | NED | 10 |
|  | 99 | 29 | 23 | IB1 | SCC | C/pL | CD/RH | ND / 0 | 35 | CD | Alive | NED | 7 |
|  | 100 | 38 | 15 | IA1 | A | C/pL | CD/TR | ND / 0 | 36 | CD | Alive | ND | ND |
|  | 101 | 34 | 22 | IB1 | SCC | C/pL/2-3 cycl Cis | CD/RH | ND / 3 | 36 | CD | Alive | NED | 5 |
|  | 102 | 38 | 7 | IB1 | A | IR/pL | RT/ChT | ND / 1 | ND | IR | Abort | ND | ND |
|  | 103 | 38 | 8 | IIA | SCC | IR/pL | RT/ChT | ND / 1 | ND | IR | Abort | ND | ND |
|  | 104 | 31 | 14 | IB1 | SCC | C/pL/2-3 cycl Cis | CD/RH | ND / 1 | 32 | CD | Alive | ND | ND |
|  | 105 | 29 | 18 | IB1 | A | C/pL/2-3 cycl Cis | CD/RH | ND / 0 | 34 | CD | Alive | ND | ND |
| Herod JJ et al., 2010 | 106 | 23 | 5 | IA2 | A | C/pL | None | 3 / 2 | 36 | CD | Alive | NED | 28 |
|  | 107 | 32 | 8 | IB1 | A | C/pL | CD/TH | 2 / 1 | 41 | CD | Alive | NED | 31 |
| Marnitz S et al., 2010 | 108 | 35 | 15 | IB1 | A | C/pL/3 cycl Cis | CD/RH | 2 / 1 | 33 | CD | Alive | NED | 17 |
|  | 109 | 31 | 20 | IB1 | SCC | C/pL/3 cycl Cis | CD/RH | 2 / 1 | 33 | CD | Alive | NED | 12 |
|  | 110 | 35 | 22 | IB1 | SCC | C/pL/3 cycl Cis | CD/RH | 4 / 3 | 36 | CD | Alive | NED | 7 |
|  | 111 | 36 | 15 | IB1 | SCC | C/pL/3 cycl Cis | CD/RH | 1 / 0 | 33 | CD | Alive | NED | 3 |
|  | 112 | 29 | 19 | IB1 | A | C/pL/3 cycl Cis | CD/RH | 1 / 0 | 34 | CD | Alive | NED | 3 |
|  | 113 | 36 | 20 | IVB | SCC | pL*/3 cycl Cis | CD/RH, RT/ChT | 2 / 1 | 35 | CD | Alive | NED | 3 |
|  | 114 | 35 | 19 | IA1 | A | C/pL/3 cycl Cis | CD/RH | 1 / 0 | 35 | CD | Alive | NED | 1 |
| Rabaiotti E et al., 2010 | 115 | 27 | 15 | IB2 | SCC | Cis | CD/RH, RT/ChT | 1 / 0 | 32 | CD | Alive | DOD | 24 |
| Hafeez I et al., 2011 | 116 | 35 | PP | IB2 | SCC | None | RH, RT | 3 / 2 | 40 | VD | Alive | RD | 5 |
| Hidaka T et al, 2011 | 117 | 26 | 10 | IA1 | SCC | C | None | ND | 37 | ND | Alive | RD | 96 |
| Li J et al., 2011 | 118 | 36 | 27 | IB2 | SCC | 2 cycl Cis/Pac | CD/RH, RT/ChT | 4 / 0 | 34 | CD | Alive | NED | 21 |
|  | 119 | 39 | 30 | IB2 | SCC | 1 cycl Cis/Pac | CD/RH | 4 / 0 | 34 | CD | Alive | NED | 13 |
| Guzel AI et al. 2013 | 120 | 47 | 5 | IB1 | SCC | IR | RH | 8 / 7 | 7 | IR | Alive | NED | ND |
| Geijteman EC et al., 2014 | 121 | 34 | 25 | IIB | SCC | Cis/Pac weekly (5 wks) | RT | 4 / 1 | 35 | CD | Alive | NED | ND |
| A, adenocarcinoma; A/SCC, adenosquamous cancer; Bleomycin, Bleo; C, conization; CD, cesarean delivery; ChT, chemotherapy; Cis, cisplatin; cycl, cycles; DOD; dead of disease; GA, gestational age; | | | | | | | | | | | | |  |
| IR, interruption; N, neuroendocrine carcinoma; NED; no evidence of disease; ND, not determined; NICU, neonatal intensive care unit; Pac, paclitaxel; | | | | | | | | |  |  |  |  |  |
| PC, preconception; pL, pelvic lymphadenectomy; PP, postpartum; RD, recurrent disease; RH, radical hysterectomy; RT, radiotherapy; | | | | | | | |  |  |  |  |  |  |
| SCC, squamous cell carcinoma; TH, total hysterectomy; TR, trachelectomy; VD, vaginal delivery; Vin, vincristine; wks, weeks; yrs, years. | | | | | | | |  |  |  |  |  |  |
| *, includes left scalene lymphnode dissection. | | |  |  |  |  |  |  |  |  |  |  |  |
| **, includes the application of cisplatin/vincristine/bleomycin and cisplatin/paclitaxel. | | | | |  |  |  |  |  |  |  |  |  |
